# An Atypical Presentation of Cutaneous Leishmaniasis

**DOI:** 10.7759/cureus.22836

**Published:** 2022-03-04

**Authors:** Fatimazahra Chahboun, Madiha Eljazouly, Maha Alj, Soumiya Chiheb

**Affiliations:** 1 Dermatology Unit, Cheikh Khalifa International University Hospital, Mohammed VI University of Health Sciences, Casablanca, MAR

**Keywords:** diabetes, tca, meglumine antimonate, verrucous, cutaneous leishmaniasis

## Abstract

Cutaneous leishmaniasis is a parasitic infection characterized by a high clinical polymorphism. Unusual clinical aspects have been reported in immunodeficient patients or associated with particular parasite species. This is the case report of a 36-year-old man with a history of type 1 diabetes who presented with a verrous plaque on the dorsal aspect of the fourth finger of the left hand, which appeared six months after traveling to southern Morocco. It was a papulo-nodular verrucous lesion, and was nonpruritic and nonpainful, with a keratotic surface, which had been progressively increasing in volume. A skin biopsy was performed, which showed evidence of leishmaniasis bodies after specific staining (May-Grünwald Giemsa stain). The patient was treated for eight weeks with weekly intralesional injections of meglumine antimoniate (Glucantime) and touch-ups with trichloroacetic acid (TCA) 20%. The evolution was marked by a clear regression of the lesion after four months. Herein, we describe a particular clinical aspect of cutaneous leishmaniasis: the verrucous form. This is a rare presentation that suggests the role of factors related to parasite species and/or diabetes. The combination of TCA with meglumine antimonate is a promising treatment option.

## Introduction

Cutaneous leishmaniasis is a parasitic infection characterized by a high clinical polymorphism. It is an endemic disease in Morocco, where its frequency has stabilized since 2010 but remains high despite the implementation of prevention and control measures [1]. Its clinical features can vary from a single chronic lesion to disseminated nodular lesions. However, several unusual and atypical disease patterns have been described related to the variability of the Leishmania species as well as the immune status of the host [2]. Herein, we report a case of cutaneous leishmaniasis with a verrucous appearance in a patient with diabetes who was treated with meglumine antimonial intralesional injections and trichloroacetic acid (TCA) dabbing.

## Case presentation

**Figure 1:**
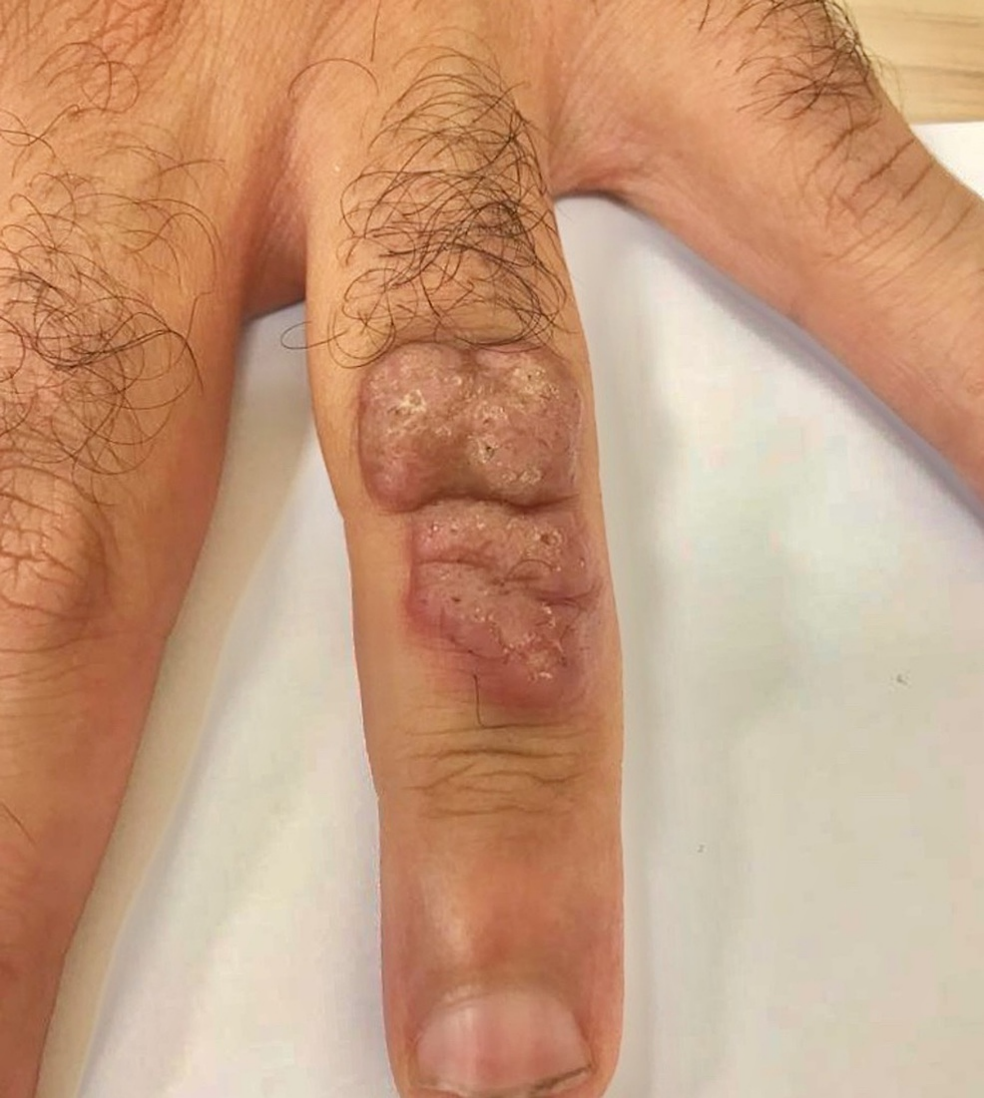
Verrucous lesion located on the dorsal aspect of the fourth finger of the left hand

The patient was a 36-year-old man with a five-year history of type 1 diabetes. History-taking revealed a stay in southern Morocco following which six months later, a proliferative lesion appeared on the back of the fourth finger of his left hand. This lesion gradually increased in volume, but it was not pruritic or painful. Physical examination revealed a verrucous lesion with a keratotic surface (Figure 1).

**Figure 2:**
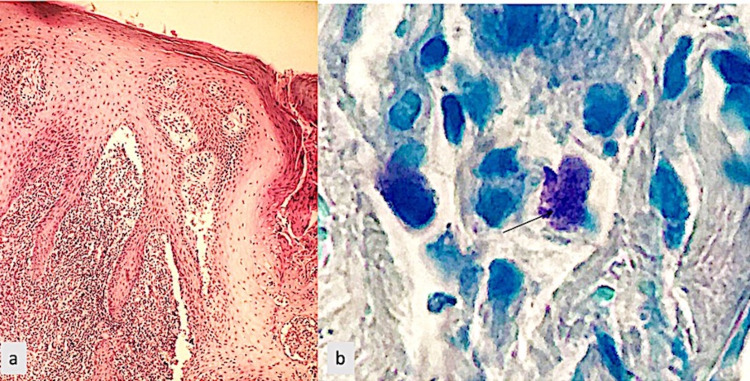
(a) Histological image of a slightly verrucous and hyperkeratotic epidermis partially ulcerated on the dermis with a lymphohistiocytic infiltrate containing macrophagic elements whose cytoplasm is occupied by foreign bodies. (b) Histological image after specific staining (May-Grünwald Giemsa stain) showing Leishmania bodies (black arrow)

Several differential diagnoses were discussed: verrucous tuberculosis, verrucous carcinoma, giant wart, cutaneous leishmaniasis, and deep mycosis. Skin biopsy with histological examination showed the presence of leishmaniasis bodies after specific staining (Figure 2).

**Figure 3:**
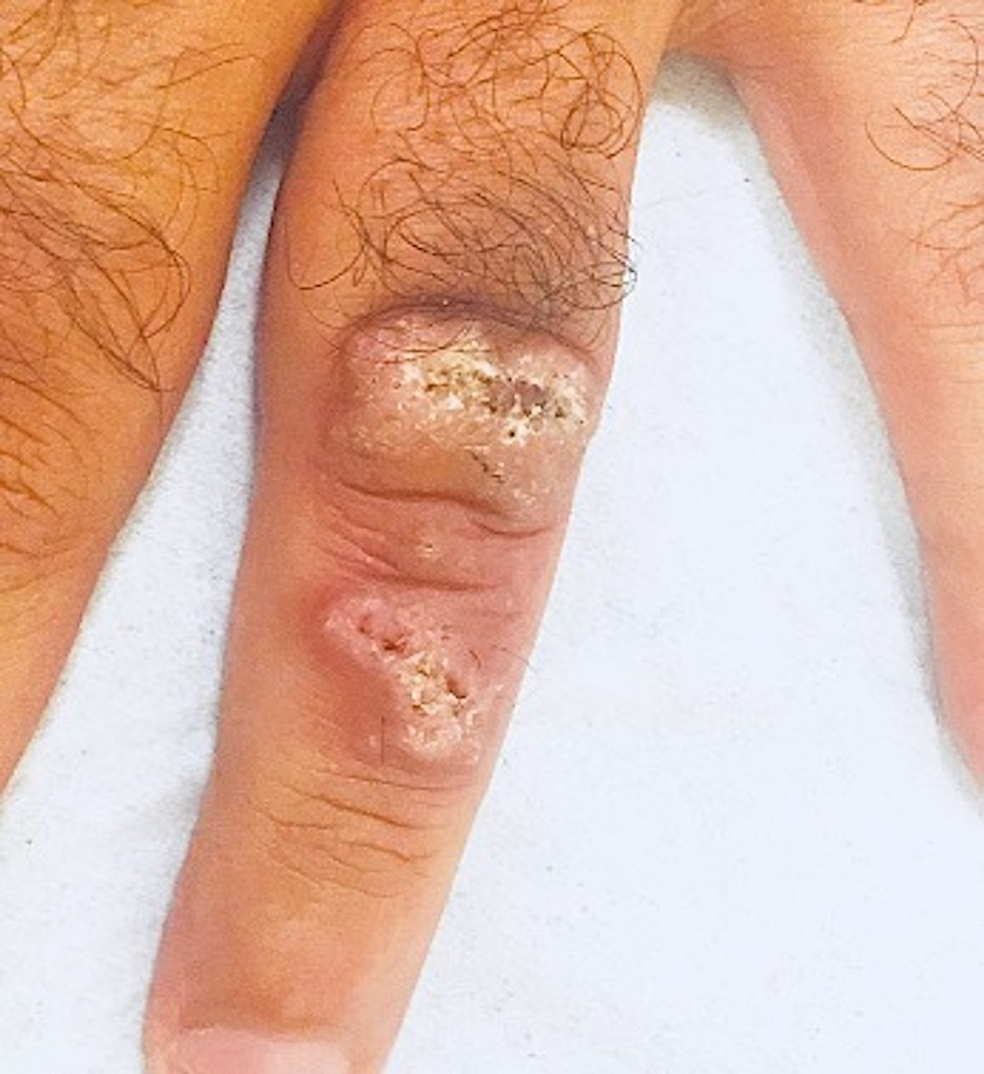
Image showing a clear regression of the lesion after the treatment

The patient was treated for eight weeks with weekly intralesional injections of meglumine antimonate (Glucantime) combined with retouches of TCA 20%. This led to a clear regression of the lesion after four months (Figure 3).

## Discussion

Cutaneous leishmaniasis is an infectious disease caused by macrophage parasitism by flagellate protozoa belonging to the genus Leishmania [2]. Several clinical presentations of cutaneous leishmaniasis have been described in the literature. The classic form is an ulcerated and nodular lesion, also known as the oriental pimple. Atypical presentations include lupoid, eczematous, erysipeloid, zosteriform, paronychia, sporotrichoid, chancriform, and annular forms. Thus, the differential diagnosis includes other infectious, inflammatory, and tumor diseases [3-7]. The verrucous form is a rare manifestation characterized by a single, rather large, well-limited plaque with a keratotic or papillomatous surface, generally located on the lower limbs. This is rarely seen on the upper limbs, as in our patient’s case.

The polymorphism of these clinical forms is often due to the different sites of the lesion. The verrucous form is the appanage of the extremities, as in the case of our patient. Polymorphism can also be caused by the infecting species, the duration of evolution of the lesion, and the inflammatory reaction elicited [2]. However, some authors have also considered the involvement of other factors in this particular lesion profile, such as an immunosuppressive state (as seen in patients with diabetes) [8,9].

The immunological response to leishmaniasis is complex and largely T-cell mediated. In Leishmania infections, the ability of macrophages to effectively kill the intracellular parasite determines the extent of disease. This usually requires the production of adequate interferon-γ (IFN-γ) by Th1 cells to activate the infected macrophages [10]. Diabetes mellitus is recognized by the World Health Organization as a cause of secondary immunodeficiency [11]. The defective host immune factors caused by diabetes mellitus in our patient may have led to a weakened T-cell response to Leishmania antigens, resulting in the unusual clinical presentation.

The diagnosis of cutaneous leishmaniasis is based on epidemiological and clinical evidence and must be confirmed by the detection of the parasite in a smear or biopsy or via polymerase chain reaction (PCR). Histopathological examination, with standard hematoxylin and eosin staining or Giemsa staining, often reveals Leishman bodies in macrophages [2]. The PCR technique is not widely available in Morocco. In our case, the patient’s travel to an endemic area, the chronicity of the lesion, its resistance to antiseptics and antibiotics, its warty appearance, and the presence of Leishman bodies on biopsy supported our diagnosis. Antimonial derivatives are the standard treatment for leishmaniasis [2].

TCA is a chemical peel initially used for skin rejuvenation, but its indications have been extended in dermatology due to its procollagenic, immunomodulatory, and keratolytic properties [12,13]. Its use in combination with meglumine antimonial intralesional injections yields better results (i.e., better cure rate with shorter time to cure) than meglumine antimonial intralesional injections alone [12].

## Conclusions

The diagnosis of cutaneous leishmaniasis should be routinely considered in the presence of any chronic lesion resistant to the usual treatments, especially for patients in endemic areas or travelers from these regions. Even if the usual clinical presentations of leishmaniasis are easily diagnosed in endemic areas, its atypical forms may be difficult to diagnose, thereby delaying appropriate treatment. TCA with meglumine antimonate is a promising treatment option for cutaneous leishmaniasis, but further studies are required to confirm this.
